# A Case of Ruptured Perineal Epidermal Cyst

**DOI:** 10.7759/cureus.11099

**Published:** 2020-10-22

**Authors:** Rahul Gupta, Piyush Verma, Nalini Bansal, Tushar Semwal

**Affiliations:** 1 Gastrointestinal Surgery, Synergy Institute of Medical Sciences, Dehradun, IND; 2 Department of Histopathology, Fortis Escorts Heart Institute, New Delhi, IND; 3 Radiology, Synergy Institute of Medical Sciences, Dehradun, IND

**Keywords:** epidermal cyst, epidermoid cyst, surgery, perineum

## Abstract

Epidermal cysts are common skin lesions. They are usually seen in the chronic sun-exposed areas of the skin. They can get complicated by inflammation, infection, rupture, or malignancy. Epidermal cyst in the perineal region is rare. We report a case of an infected ruptured perineal epidermal cyst mimicking gluteal abscess. The patient was successfully treated by complete surgical excision. Histology revealed epidermal cyst with foreign body giant cell reaction and no evidence of malignancy.

## Introduction

Epidermal cysts are benign slow-growing lesions derived from the epidermal layer of the skin [1]. They develop as a result of proliferation of epidermal cells within the dermis. These cysts contain keratin and are lined by stratified squamous epithelium [2]. These are more commonly observed in males than in females and occur mostly in the fourth decade of life. These are also called epidermoid or epidermal inclusion or sebaceous cyst [2]. However, the sebaceous gland is not involved in epidermal cysts. Thus, sebaceous cyst is a misnomer. These lesions may remain stable or progressively get enlarged. These can occur in any part of the body but are seen more often in chronic sun-exposed areas [1]. Presence of epidermal cyst in the perineal region is quite rare [1,3-6]. We report a case of a large perineal epidermal cyst with spontaneous rupture treated successfully by surgical excision.

## Case presentation

**Figure 1:**
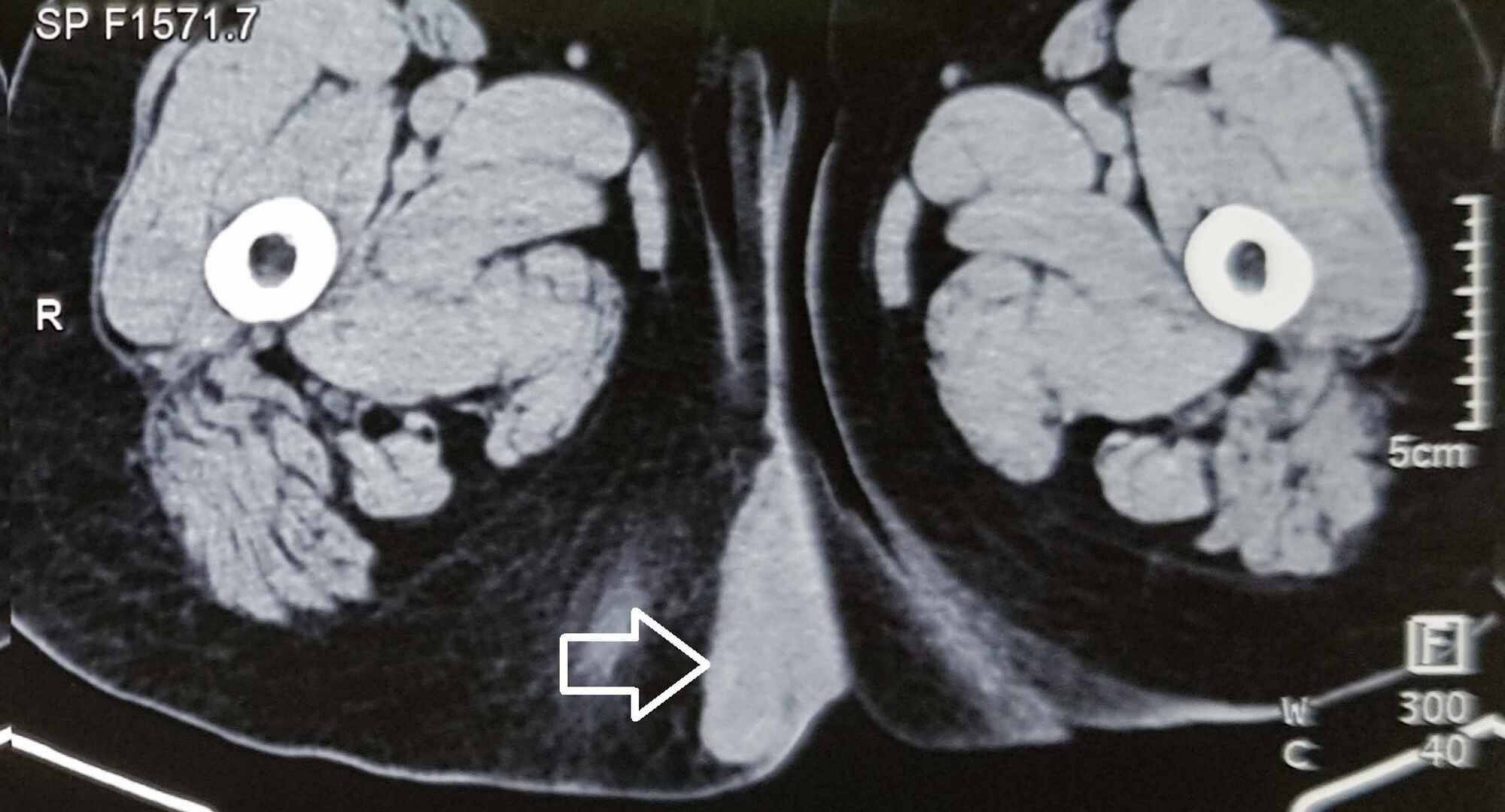
Computed tomography of the pelvis showing the perineal lesion suggestive of abscess formation (arrow).

A 61-year-old normotensive and euglycemic female presented with complaints of painful swelling in the right gluteal and perineal region with purulent discharge for one week. She also had fever for three days. She noticed an ill-defined swelling in the perineal region many months back without any symptoms. The swelling suddenly became painful and started discharging fluid for one week. There was no associated history of bleeding per rectum or constipation. She has past history of cholecystectomy and hysterectomy done three and 10 years back, respectively. On physical examination, there was a soft swelling of 5 x 5 cm in the perineal region on the right side. The swelling was located in the subcutaneous plane with an external opening at its summit discharging yellowish-white fluid. There was no induration, tenderness or redness over the swelling. Contrast enhanced computed tomography (CECT) of the abdomen and pelvis revealed a well-defined 6.4 (anteroposterior) x 1.7 (transverse) x 5.2 (craniocaudal) cm, irregularly marginated hypodense (HU20-35) lesion present in the subcutaneous plane of right gluteal region in the paramedian location (Figure 1). 

No obvious calcification or solid component was seen. There was mild adjacent subcutaneous fat stranding with no obvious enhancement or intralesional vascularity after intravenous contrast injection. Superior-medially it was seen reaching up to the level of anal verge on the right side. However, no obvious communication with distal rectum or anus was seen. The final diagnosis of right gluteal abscess was made on CECT. In view of above findings, surgery was planned.

**Figure 2:**
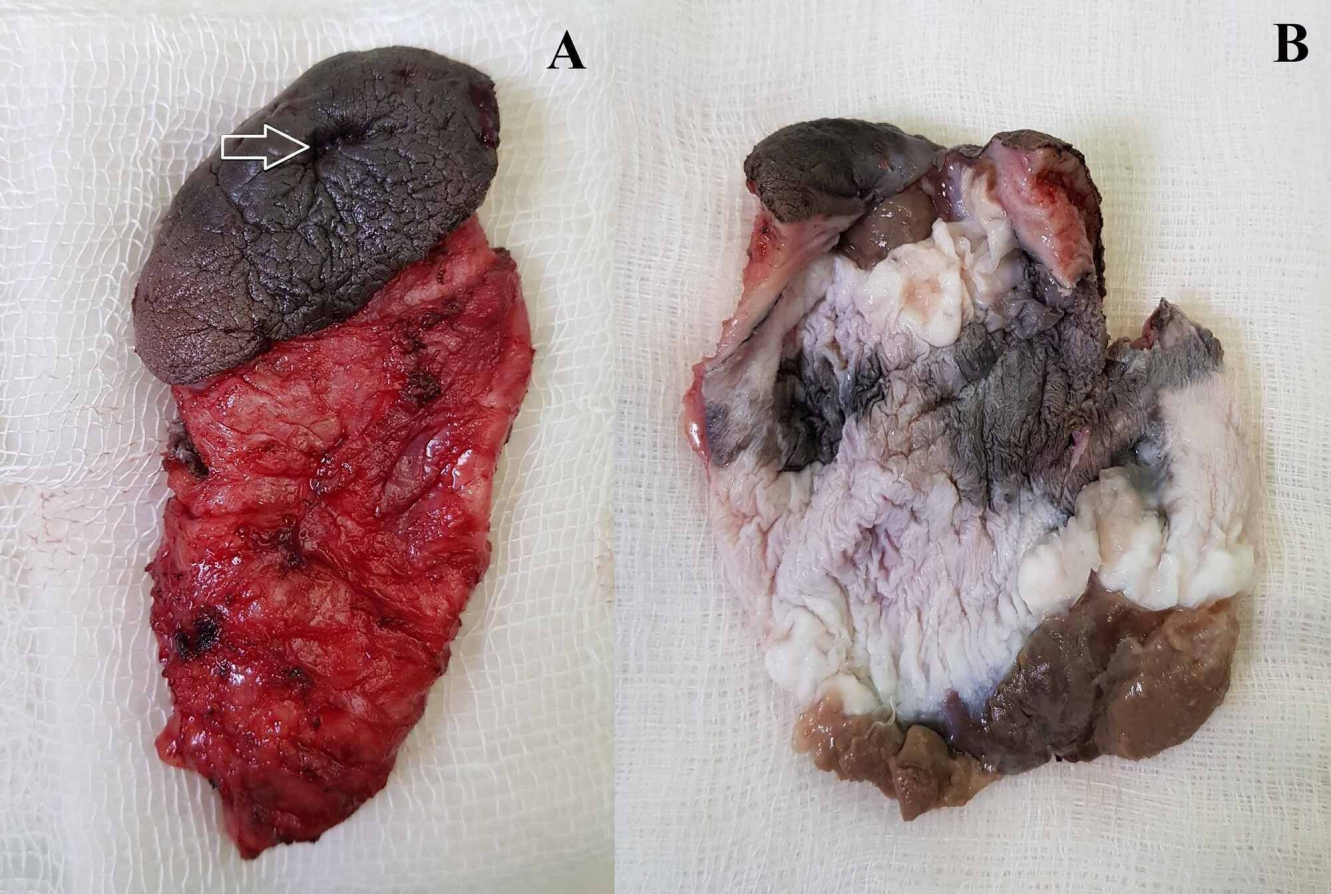
Ruptured perineal cyst: (A) resected specimen showing the external opening (arrow), (B) cut section showing the cyst lined by thick, multicolored epithelium. No mural nodules were observed.

The patient was administered spinal anesthesia and placed in lithotomy position. Intraoperatively, there was a large ruptured cyst of 7 x 7 x 6 cm in the right perineal region about 3-4 cm lateral to the anal opening. The cyst was thick walled containing sebaceous material and was located in the subcutaneous plane. Simple excision of the ruptured perineal cyst was done and the wound was primarily sutured. The resected specimen was sent for histopathology (Figure 2). 

**Figure 3:**
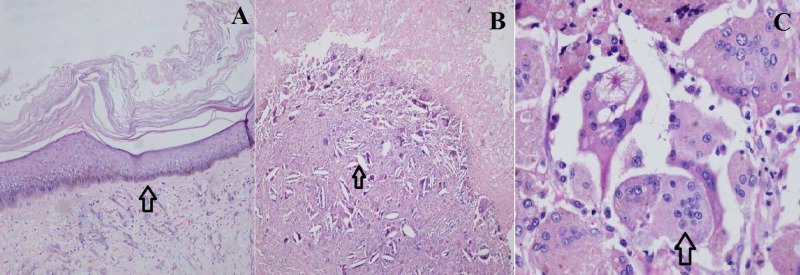
Microscopic examination of the perineal cyst showing the cyst lined by keratinized stratified squamous epithelium (arrow) (H&E, 10x) (A), presence of subepithelial granular layer containing cholesterol clefts (arrow) (H&E, 40x) (B) and foreign body giant cells (arrow) (H&E, 100x) (C). H&E: Hematoxylin and eosin

In the postoperative follow-up, the patient developed wound infection which was treated by dressings and oral antibiotics. On cut section, the cyst was filled with sebaceous material and lined by thick multicolored epithelium (Figure 2). On microscopic examination, the cyst was lined by keratinized squamous epithelium with the presence of granular layer in the subepithelial region (Figure 3). 

The cyst lumen contained lamellated keratinous material. The cyst was disrupted at places with presence of foreign body giant cell reaction and cholesterol clefts (Figure 3). The final diagnosis of benign inflamed epidermal cyst was made. On the last follow-up at nine months, the patient was disease-free.

## Discussion

An epidermal cyst is caused by the invagination of the epidermis into the subcutaneous layer from a hair follicle [7]. The most common type of epidermal cyst is a follicular infundibular cyst. Several factors may be responsible for epidermal cyst formation, including exposure to ultraviolet rays, human papilloma virus infection, smoking, and trauma (surgical cuts such as episiotomy or needle biopsies in the perineal region) [1,8,9]. Multiple epidermoid cysts can also be found in autosomal dominant Gardner syndrome (familial adenomatous polyposis) or Gorlin syndrome (basal cell nevus syndrome) [4]. Epidermal cysts are usually asymptomatic. However, they may become secondarily inflamed or infected or rarely develop malignancy [5]. There are very few reported cases of cutaneous squamous cell carcinoma (SCC) arising within epidermal cysts [10]. SCC within perineal epidermal cysts is even more rare [5]. 

Epidermal cysts are most commonly located on the head and neck but limbs, trunk and perineal region including scrotum and genitalia can also be affected [10]. An epidermal cyst may become ruptured depending upon the location and chances of external pressure. They are usually movable and typically produce symptoms of inflammation when infected. They may produce discomfort when their size increases especially in the perineal region. Differential diagnoses of epidermal cyst in the perineal region are lipoma, abscess, pilonidal cyst, dermoid cyst, soft tissue tumor such as benign teratoma, and skin cancer [1]. Clinically, it is often difficult to distinguish between benign and malignant cystic lesions. In the perineum, it is important to differentiate between an abscess, a common clinical condition, and infected perineal cyst, an uncommon disease because incision and drainage are sufficient for abscess while complete excision is required for infected perineal cyst. Radiological investigations can help in determining the nature of the perineal swelling. In doubtful cases, fine needle aspiration and/or biopsy can be performed to determine the histology of the lesion.

On magnetic resonance imaging (MRI), the cyst shows low and intermediate/high signal intensity on T1-weighted and T2-weighted images, respectively. The cyst has high signal intensity on fat-saturated images, thin peripheral enhancement, and significant diffusion restriction [1]. On CT, cysts appear as well-defined hypodense lesions with a capsule [3]. Any contrast-enhancing mural nodule or focal thickening of the wall should raise the suspicion of malignancy.

The treatment for epidermal cyst is simple excision. The epidermal cyst of the perineum can be excised under local, saddle, or spinal anesthesia. If malignancy is suspected preoperatively, then wide local excision should be performed. Histological examination of the resected specimen is important to detect squamous cell carcinoma if present.

## Conclusions

Epidermal cyst of the perineal region may be found in patients with perineal trauma due to any past surgery or chronic hair follicle irritation mimicking abscess. Plugging of the hair follicle orifice may trigger its development. It should be included in differential diagnosis while treating patients with perineal swelling. It can be complicated by infection, inflammation, or malignancy. Inflammation of the cyst usually results in its rupture. Timely surgical intervention is needed whenever it enlarges to produce discomfort or gets inflamed.
